# Effect of Surgical Day of Week on Postoperative Outcomes After Robotic-Assisted Pulmonary Lobectomy

**DOI:** 10.7759/cureus.35379

**Published:** 2023-02-23

**Authors:** Hudson Ash-Rafzadeh, Tilman J Chambers, Frank O Velez, Carla C Moodie, Joseph R Garrett, Jacques P Fontaine, Eric M Toloza

**Affiliations:** 1 Department of Medical Education, University of South Florida Health Morsani College of Medicine, Tampa, USA; 2 Department of Surgery, University of South Florida Health Morsani College of Medicine, Tampa, USA; 3 Department of Thoracic Oncology, Moffitt Cancer Center, Tampa, USA; 4 Departments of Surgery and of Oncologic Sciences, University of South Florida Health Morsani College of Medicine, Tampa, USA

**Keywords:** perioperative outcomes, thoracic oncosurgery, patient-centered outcomes research, pulmonary lobectomy, robotic surgical procedures

## Abstract

Introduction

Patients who have surgery late in the week could potentially receive different postoperative care due to a reduced weekend staff compared to patients who have surgery early in the week, who will be cared for by a full staff during the work week. Our aim was to determine if patients who underwent robotic-assisted video-thoracoscopic (RAVT) pulmonary lobectomy during the first half of the week had different outcomes than patients who also underwent RAVT pulmonary lobectomy during the second half of the week.

Methods

We analyzed 344 consecutive patients who underwent RAVT pulmonary lobectomy by one surgeon from 2010 to 2016. Depending on the day of the surgical procedure, these patients were either put into a Monday through Wednesday (M-W) group or a Thursday through Friday (Th-F) group. Patient demographics, tumor histopathology, intraoperative and postoperative complications, and perioperative outcomes were compared between groups using the Student’s t-test, Kruskal-Wallis test, or chi-square (or Fisher’s exact) test, with p≤0.05 as significant.

Results

There were more non-small cell lung cancers (NSCLCs) resected in the M-W group than in the Th-F group (p=0.005). Skin-to-skin and total operative times were greater for the Th-F group than for the M-W group (p=0.027 and p=0.017, respectively). There were no significant differences in any other variables assessed.

Conclusions

Our study showed that, despite reduced weekend staffing and potential differences in postoperative care, there were no significant differences seen in postoperative complications or perioperative outcomes based on surgical day of the week.

## Introduction

The logistics surrounding hospital staffing can be challenging, especially on weekends. Patients who have surgery on Thursday or Friday receive their postoperative care during the weekend, which may be staffed less than during the week. This difference in staffing brings the potential for patients to receive different levels of postoperative care based on the day of the week on which a patient had their surgical procedure. An association between weekend care and poor outcomes has become known as the “weekend effect,” with studies showing rises in postoperative mortality as the day of elective surgery approaches the weekend as well as higher mortality rates for emergency surgeries done on the weekends compared to weekdays [1,2]. The first 48 h after surgery is a crucial period, and admission to the surgical intensive care unit (ICU) can decrease postoperative mortality [3]. On the other hand, weekend hospital admissions, including ICU admissions, have shown to be associated with higher mortalities, which could be attributed to a lower level of staffing and intensity of care over the weekends [4-6]. Furthermore, the “weekend effect” seems to be a systemic phenomenon affecting healthcare systems globally [7]. However, the specific effects involving the specific day of the week on postoperative complications and outcomes for elective robot-assisted pulmonary lobectomies have not been well studied.

The purpose of this study was to assess if patients who underwent surgery late in the week had different outcomes than patients who had surgery earlier in the week. Patients who had surgery later in the week could potentially receive different postoperative care due to reduced weekend staff compared to patients who had surgery earlier in the week and who had postoperative care by a full staff during the work week. If there were differences in outcomes, hospital staffing for weekend care would need to be adjusted accordingly.

This study was previously presented in part at the 14th Annual Academic Surgical Congress in Houston, TX, USA, on February 6, 2019.

## Materials and methods

We retrospectively identified 344 consecutive patients who underwent robotic-assisted video-thoracoscopic (RAVT) pulmonary lobectomy from 2010 to 2016 by one surgeon at a single cancer center. Patients were grouped into those who had surgery on Monday, Tuesday, or Wednesday (M-W group) versus those who had surgery on Thursday or Friday (Th-F group). Our RAVT pulmonary lobectomy approach and technique have been described in detail previously [8].

Patient demographics, tumor histopathology, intraoperative and postoperative complications, and perioperative outcomes were analyzed. A wide variety of intraoperative and postoperative complications were analyzed with a liberal definition of complications including any findings or events that could alter the clinical course or ultimate outcomes. Postoperative complications were further grouped into pulmonary and cardiovascular categories.

Demographics, tumor histopathology, and intraoperative and postoperative complications are reported as mean with standard error of the mean or as number count and percentage, as appropriate. Intraoperative estimated blood loss (EBL), skin-to-skin operative time, chest tube duration, hospital length of stay (LOS), and in-hospital mortality were also evaluated as perioperative outcomes, with intraoperative EBL, skin-to-skin operative time, chest tube duration, and hospital LOS each being reported as a median and the first quartile (Q1) and third quartile (Q3) values of the range. Variables and outcomes were compared between groups using Student’s independent samples t-test, Kruskal-Wallis test, Pearson chi-square analysis, or Fisher’s exact test, as appropriate, with significance at p≤0.05.

## Results

There were 344 total patients in the study cohort, with 182 patients in the M-W group and 162 patients in the Th-F group. Table 1 shows the demographics of the study population. Median age was 67 years (range: 24-87 years). Women comprised 59% (n=202) of the patients.

**Table 1:** Patient’s demographic and tumor characteristics. *Mean±SEM (range). SEM: standard error of the mean; M-W: date of surgery on Monday, Tuesday, or Wednesday; Th-F: date of surgery on Thursday or Friday; BSA: body surface area; BMI: body mass index; NSCLC: non-small cell lung cancer; path: pathologic

Variables			Total	M-W	Th-F	p-Value
			N=344	n=182	n=162	
Age* (years)			67.6±1.1 (24-87)	67.9±0.7 (24-86)	67.3±0.8 (28-87)	0.400
Gender		Male	142 (41.2%)	77 (43%)	65 (40%)	0.740
		Female	202 (58.7%)	105 (57%)	97 (60%)	
BSA* (m^2^)			1.87±0.3 (1.2-2.9)	1.87±0.20 (1.2-2.9)	1.88±0.20 (1.3-2.9)	0.580
BMI* (kg/m^2^)			27.6±4.3 (14-59)	27.3±0.4 (14-59)	28.0±5.8 (16.8-55)	0.170
Tumor size (cm)		All	3.3±0.1 (0.5-14.2)	3.3±0.2 (0.5-14.2)	3.3±0.2 (0.8-9.7)	0.829
		NSCLC	3.3±0.1 (0.5-14.2)	3.3±0.2 (0.5-14.2)	3.3±0.2 (0.8-9.7)	0.809
Tumor type	NSCLC		310 (90.1%)	172 (95%)	138 (85%)	0.005*
	Other		34 (9.9%)	10 (5%)	24 (15%)	
NSCLC path stages	Total		N=310	n=172	n=138	0.463
	IA		137 (44.2%)	74 (43%)	63 (45.7%)	
	IB		40 (12.9%)	20 (11.6%)	20 (14.5%)	
	IIA		42 (13.5%)	21 (12.2%)	21 (15.2%)	
	IIB		21 (6.8%)	15 (8.7%)	6 (4.3%)	
	IIIA		56 (18.1%)	35 (20.4%)	21 (15.9%)	
	IIIB		4 (12.9%)	1 (0.6%)	3 (2.2%)	
	IV		9 29.0%)	6 (3.5%)	3 (2.2%)	

There were significantly (p=0.005) more non-small cell lung cancers (NSCLC) resected in the M-W group (n=172) than in the Th-F group (n=138) (Table 1). Tumor size and histologic subtype were analyzed, and the most common histologies were adenocarcinoma (61.0%), squamous cell carcinoma (17.7%), and neuroendocrine carcinomas (6.7%). There were no significant differences in tumor size or histology between groups (p=0.829 and p=0.584, respectively) (Tables 1, 2).

**Table 2:** Tumor histology. M-W: date of surgery on Monday, Tuesday, or Wednesday; Th-F: date of surgery on Thursday or Friday

Tumor type	Total	M-W	Th-F	p-Value
Total	N=344	n=182	n=162	0.584
Adenocarcinoma	210 (61.0%)	116 (63.4%)	94 (57.9%)	
Squamous	61 (17.7%)	36 (19.6%)	25 (15.4%)	
Neuroendocrine	23 (6.7%)	10 (5.4%)	13 (8%)	
Mixed tumors	17 (4.9%)	11 (5.8%)	6 (3.6%)	
Small cell	4 (1.2%)	0 (0%)	4 (2.5%)	
Metastases	25 (7.3%)	8 (4.1%)	17 (10.3%)	
Lymphoma	1 (0.3%)	0 (0%)	1 (0.6%)	
Benign lesions	3 (0.9%)	1 (0.5%)	2 (1.2%)	

After analysis of each of the clinical variables, 183 patients (53%) experienced postoperative complications, with the majority (39%) being pulmonary complications, while only 25 patients (7%) had intraoperative complications. There were no significant differences in rates of intraoperative or postoperative complications between the two groups (Tables 3, 4).

**Table 3:** Intraoperative complications. M-W: date of surgery on Monday, Tuesday, or Wednesday; Th-F: date of surgery on Thursday or Friday

Variables	Total	M-W	Th-F	p-Value
	N=344	n=182	n=162	
Overall intraoperative complications	26 (7.3%)	15 (8.2%)	11 (6.8%)	0.51
Bleeding	16 (4.7%)	9 (5.0%)	7 (4.3%)	0.90
Phrenic nerve injury	1 (0.3%)	1 (0.5%)	0 (0%)	0.40
Recurrent laryngeal nerve injury	3 (0.9%)	1 (0.5%)	2 (1.2%)	0.40
Bronchial injury	5 (1.5%)	3 (1.6%)	2 (1.2%)	0.07
Diaphragm injury	1 (0.3%)	1 (0.5%)	0 (0%)	0.40
Robotic-related intraoperative complications	19 (5.5%)	13 (7.1%)	6 (3.7%)	0.76
Overall conversion to open lobectomy	26 (7.6%)	17 (9.3%)	9 (5.6%)	0.78
Urgent conversion due to bleeding	11 (3.2%)	5 (2.7%)	6 (3.7%)	0.81

**Table 4:** Postoperative complications. *Some patients had more than one complication. M-W: date of surgery on Monday, Tuesday, or Wednesday; Th-F: date of surgery on Thursday or Friday; MOSF: multiorgan system failure

Variables	Total N=344	M-W	Th-F	p-Value
		n=182	n=162	
Overall postoperative complications*	183 (53.2%)	106 (58.2%)	77 (47.5%)	0.31
Pulmonary complications*	134 (39.0%)	76 (41.8%)	58 (35.8%)	0.47
Prolonged air leak for ≥seven days	62 (18.0%)	38 (20.9%)	24 (14.8%)	0.16
Pneumothorax	6 (1.7%)	4 (2.2%)	2 (1.2%)	0.69
Mucous plug	12 (3.5%)	7 (3.8%)	5 (3.1%)	0.78
Pneumonia	19 (5.5%)	10 (5.5%)	9 (5.6%)	0.14
Chyle leak	11 (3.2%)	7 (3.8%)	4 (2.5%)	0.55
Hemothorax	4 (1.2%)	1 (0.5%)	3 (1.9%)	0.35
Pulmonary embolism	2 (0.6%)	0 (0%)	2 (1.2%)	0.22
Other pulmonary complications	20 (5.8%)	10 (5.5%)	10 (6.2%)	0.83
Cardiovascular complications*	45 (13.1%)	28 (15.4%)	17 (10.5%)	0.21
Atrial fibrillation	32 (9.4%)	18 (9.9%)	14 (8.6%)	0.71
Other arrhythmias	4 (1.2%)	2 (1.1%)	2 (1.2%)	1.00
Cerebrovascular accident	1 (0.3%)	1 (0.5%)	0 (0%)	1.00
Myocardial infarction	2 (0.6%)	1 (0.5%)	1 (0.6%)	1.00
Shock/MOSF	4 (1.2%)	3 (1.6%)	1 (0.6%)	0.63
Cardiopulmonary arrest	3 (0.9%)	3 (1.6%)	0 (0%)	0.25

On analysis of perioperative outcomes, the Th-F group had a median skin-to-skin operative time 22 minutes longer than that of the M-W group (188 minutes versus 166 minutes; p=0.027) (Table 5). In-hospital mortality was 1.2% of patients (n=4), with no intraoperative deaths.

**Table 5:** Perioperative outcomes. *Median (Q1, Q3) Q1: first quartile values; Q3: third quartile values; M-W: date of surgery on Monday, Tuesday, or Wednesday; Th-F: date of surgery on Thursday or Friday; EBL: estimated blood loss; LOS: length of stay

Variables	Total N=344	M-W	Th-F	p-Value
		n=182	n=162	
Intraoperative EBL* (mL)	157 (100, 275)	150 (100, 250)	163 (100, 300)	0.099
Skin-to-skin operative time* (minutes)	177 (143, 221)	166 (141, 211)	188 (145, 230)	0.027*
Total operative time* (minutes)	216 (180, 258)	202 (178, 248)	229 (181, 267)	0.017*
Chest tube duration* (days)	4 (3, 6)	4 (3, 6)	4 (2, 5)	0.358
Hospital LOS* (days)	4 (3, 7)	4 (3, 7)	4 (3, 6)	0.805
In-hospital mortality, n (%)	4 (1.2%)	3 (1.6%)	1 (0.6%)	0.625

## Discussion

Healthcare quality and delivery have been a topic of interest in recent decades. Patient morbidity and mortality are among the most used variables that currently exist for the evaluation of the quality of our health delivery systems [3,5-7].

The logistics surrounding staffing can be challenging. There are always concerns about whether patients are receiving different levels and quality of care based on when their surgery occurs and the availability of postoperative care staff. Patients who have surgery late in the week could receive different postoperative care due to a reduced weekend staff compared to those who have surgery earlier in the week and who receive postoperative care with a full staff [1,2,4]. This is an important question to address, because, if there are differences in quality and outcomes of care based on the timing of surgery and hospital staffing, then there would need to be adjustments in staffing for weekends.

Although there are not many studies available for a direct comparison, our results point to some interesting findings. NSCLC tumors were more common in the M-W group, although there was no conscious selection of patients with these tumors during scheduling of their surgical procedures according to preoperative diagnosis. Interestingly, these NSCLC tumors took less time to resect. These differences in perioperative outcomes, such as the skin-to-skin operative time, could be attributed to differences in tumor types requiring variable levels of dissection.

Several studies have shown the potential for poor outcomes and increase in mortality rates with admissions and surgical procedures later in the week and over the weekends, which is known as the “weekend effect” [1,2]. However, our study demonstrated otherwise. Even with differences in staffing between the weekdays and the weekends, including less staff, often with less seniority and experience, as well as lower intensity of care over the weekends, there were no significant differences in postoperative complications or perioperative outcomes in patients undergoing elective RAVT pulmonary lobectomies based on the day of the week for the surgical procedure. Furthermore, surgeons’ workloads, such as total hours of operating time and level of experience, have been shown to be associated with an increased risk of complications when performing pulmonary lobectomies [9]. Despite the expectation of seeing different outcomes in surgeries performed later in the work week by the same surgeon and surgical team, no significant differences in complications were observed in our study.

Our study is limited by being a retrospective study. Patient outcomes can be influenced by more than one factor, and understanding true causality is complicated. There are also many confounding factors that are difficult to assess in a study such as this with a relatively small sample size. The relationship between surgical day of the week and perioperative outcomes should be studied in more detail and with larger patient cohorts. This study was also limited by all patients having had their RAVT lobectomy performed by one surgeon and at one institution. While our findings reaffirm previously published reports demonstrating improved outcomes in a high-volume academic center that performs high-risk surgery and that likely has a staffing level far more experienced and familiar with complex post-operative patients than a private community hospital, the conclusions cannot be extrapolated to other institutions and may not translate to other institutions of lesser volume or those less experienced in postoperative care for thoracic patients. This investigation should be extended to other institutions and should be performed in a community setting. Indeed, if a similar study done in a community setting shows a difference in outcomes based on the day of the surgical procedure, the findings would be valuable for patients and their decision-making process. Furthermore, while our study exclusively analyzed patients who underwent RAVT pulmonary lobectomies, future studies should be done to confirm if our findings in this study are present with other surgical procedures performed via different surgical approaches by multiple surgeons across multiple institutions.

## Conclusions

The level and quality of care on the weekdays versus the weekends and their effects on outcomes and mortality rates have been the subject of study for many years. Even though this “weekend effect” has been reported, we saw no significant differences in postoperative complications after RAVT pulmonary lobectomy based on the day of the surgical procedure. This result points us to draw the conclusion that, at our cancer center, pulmonary lobectomy outcomes remain stable despite potential differences in staffing and level of postoperative care related to the day of the surgical procedure. However, there are other variables associated with postoperative complications and outcomes, and the relationship among these factors needs to be better understood with larger-scale studies on a multi-institutional level. Nevertheless, RAVT pulmonary lobectomy performed just before a weekend is feasible and safe.
